# RAGE mediates airway inflammation via the HDAC1 pathway in a toluene diisocyanate-induced murine asthma model

**DOI:** 10.1186/s12890-022-01832-3

**Published:** 2022-02-11

**Authors:** Xianru Peng, Minyu Huang, Wenqu Zhao, Zihan Lan, Xiaohua Wang, Yafei Yuan, Bohou Li, Changhui Yu, Laiyu Liu, Hangming Dong, Shaoxi Cai, Haijin Zhao

**Affiliations:** Chronic Airways Diseases Laboratory, Department of Respiratory and Critical Care Medicine, Nanfang Hospital, Southern Medical University, Guangzhou, 510515 China

**Keywords:** Toluene diisocyanate (TDI), Asthma, Histone deacetylase 1 (HDAC1), Receptor for advanced glycation end products (RAGE)

## Abstract

**Background:**

Exposure to toluene diisocyanate (TDI) is a significant pathogenic factor for asthma. We previously reported that the receptor for advanced glycation end products (RAGE) plays a key role in TDI-induced asthma. Histone deacetylase (HDAC) has been reported to be important in asthmatic pathogenesis. However, its effect on TDI-induced asthma is not known. The aim of this study was to determine the role of RAGE and HDAC in regulating airway inflammation using a TDI-induced murine asthma model.

**Methods:**

BALB/c mice were sensitized and challenged with TDI to establish an asthma model. FPS-ZM1 (RAGE inhibitor), JNJ-26482585 and romidepsin (HDAC inhibitors) were administered intraperitoneally before each challenge. In vitro, the human bronchial epithelial cell line 16HBE was stimulated with TDI-human serum albumin (TDI-HSA). RAGE knockdown cells were constructed and evaluated, and MK2006 (AKT inhibitor) was also used in the experiments.

**Results:**

In TDI-induced asthmatic mice, the expression of RAGE, HDAC1, and p-AKT/t-AKT was upregulated, and these expressions were attenuated by FPS-ZM1. Airway reactivity, Th2 cytokine levels in lymph supernatant, IgE, airway inflammation, and goblet cell metaplasia were significantly increased in the TDI-induced asthmatic mice. These increases were suppressed by JNJ-26482585 and romidepsin. In addition, JNJ-26482585 and romidepsin ameliorated the redistribution of E-cadherin and β-catenin in TDI-induced asthma. In TDI-HSA-stimulated 16HBE cells, knockdown of RAGE attenuated the upregulation of HDAC1 and phospho-AKT (p-AKT). Treatment with the AKT inhibitor MK2006 suppressed TDI-induced HDAC1 expression.

**Conclusions:**

These findings indicate that RAGE modulates HDAC1 expression via the PI3K/AKT pathway, and that inhibition of HDAC prevents TDI-induced airway inflammation.

**Supplementary Information:**

The online version contains supplementary material available at 10.1186/s12890-022-01832-3.

## Introduction

Toluene diisocyanate (TDI), a chemical used in many industries, and is a common cause of occupational asthma (OA). Epidemiological studies have shown that OA accounts for 10% to 25% of new-onset adult asthma cases [1].

The receptor for advanced glycation end products (RAGE) is a cell surface receptor belonging to the immunoglobulin superfamily that recognizes pathogen-derived endogenous ligands and initiates immune responses [2]. RAGE is most highly expressed in lung tissue, and plays an important role in the pulmonary inflammatory response [3, 4]. It has also been reported that RAGE is an important mediator of allergic inflammation and airway hyper-responsiveness (AHR) in house dust mite (HDM) and fungal extract-induced murine asthma models [5–7]. Our previous studies showed that the expression of RAGE and its ligands were increased in TDI-induced asthmatic mice, and that blocking the RAGE signal reduced airway inflammation [8–10]. However, the mechanisms underlying these effects have not was been determined.

Histone deacetylation has been reported to be important in the pathogenesis of asthma [11–13]. Histone deacetylases (HDACs) are classified into four major groups based on their homology with yeast orthologs [13]. HDAC activity is different between asthmatics and non-asthmatics, and activity is associated with corticosteroid response [14–16]. In addition, studies using ovalbumin (OVA)-induced asthma models have found that HDAC inhibitors exhibit therapeutic effects [17, 18]. However, the role of HDAC in TDI-induced asthma requires further study.

RAGE is necessary to accumulate group 2 innate lymphoid cells (ILC2s) in the lungs, contributing to allergic airway inflammation [5]. A murine model study found that the HDAC inhibitor trichostatin A also suppressed allergic inflammation by blocking ILC2 activation [19]. Besides, hyperglycemia increased the expression levels of RAGE and HDAC2 in AC16 cardiomyocytes [20]. Another study found that administration of the RAGE agonist glycated albumin increased HDAC activity in the retinal pigment epithelium [21]. However, no similar study examined the relationship between RAGE and HDAC in asthma.

Based on previous research, we hypothesized that RAGE regulates airway inflammation via the HDAC pathway. Thus, this study aimed to determine the regulatory role of RAGE on airway inflammation using a murine TDI-induced asthma model.

## Materials and methods

### Reagents

TDI (toluene-2, 4-diisocyanate), acetone, and methacholine were obtained from Sigma-Aldrich (Shanghai, China). TDI was dissolved in a mixture of acetone and olive oil (AOO): the ratio of acetone to olive oil was 2:3 for sensitization, and 1:4 for challenge. FPS-ZM1 (RAGE inhibitor), JNJ-26482585 (HDAC inhibitor), romidepsin (HDAC inhibitor), and MK2206 (AKT inhibitor) were purchased from Selleck (SelleckChem, Shanghai, China). JNJ-26482585 is a novel second-generation HDAC inhibitor with the greatest effect for HDAC1, and romidepsin is a selective inhibitor of HDAC1 and HDAC2. Anti-RAGE (ab37647), anti-HDAC1 (ab109411), and anti-β-catenin (ab32572) were purchased from Abcam. Anti-E-cadherin (#3195), anti-total-AKT (#4691), and Phospho-AKT(#4060) were obtained from Cell Signaling Technology (Boston, MA, USA).

### *Animals and treatment with RAGE and HDAC inhibitors *in vivo

All animal experiments are in accordance with the requirements of the Committee for the Use and Care of Laboratory Animals of Southern Medical University and performed under standard guidelines for the Care and Use of Laboratory Animals. Male BALB/c mice (6–8 weeks old, 20–24 g) were obtained from Southern Medical University. Mice were placed in a specific pathogen-free (SPF) environment, where they were exposed to dark–light cycle for 12 h at 24 °C in an atmosphere of 40–70% humidity and given free access to food and water. All experiments involving animals complied with the ARRIVE guidelines.

A total of 50 mice were randomly divided into the following groups, with 10 mice in each group: (1) AOO; (2) TDI; (3) TDI + FPS-ZM1; (4) TDI + JNJ-26482585; (5) TDI + romidepsin. TDI-induced murine asthma models were established as previously described [8]. On day 1 and day 8, the mice were dermally sensitized with 0.3% TDI (20 µL per ear) on the dorsum of both ears. On day 15, 18, and 21, the mice were separately placed in a horizontal cylindrical chamber for an airway challenge with 3% TDI dissolved in acetone/olive oil (1:4). The TDI was dispersed by compressed air nebulization (NE-C28; Omron, Tokyo, Japan), and the mice remained in the chamber for 3 h. The control mice were sensitized and challenged by the same method using the same volume of AOO rather than TDI. Before each challenge, mice were respectively injected with FPS-ZM1 (1.5 mg/kg, intraperitoneally [i.p.]), JNJ-26482585 (5 mg/kg, i.p.), and romidepsin (2.4 mg/kg, i.p.), which were dissolved in DMSO and diluted with PBS. The control group received the same amount of vehicle.

### Assessment of AHR

AHR to methacholine was assessed by measuring lung resistance (R_L_) (Buxco Electronics, Troy, NY) on day 22, using the method previously described [22]. Mice in each group were placed in a barometric plethysmographic chamber and then challenged with sterile saline, followed by increasing concentrations of aerosolized methacholine (3.125, 6.25, 12.5, and 25 mg/mL). R_L_ was recorded every 5 min following each nebulization step according to the manufacturer’s protocol. R_L_ was recorded as the percentage of baseline value (value for sterile saline) for each concentration of methacholine.

### Measurement of interleukin (IL)-4, IL-5, and IL-13 in supernatants of cultured lymphocytes

The cervical lymph nodes of mice were isolated and pressed into a 40 µm cell strainer (BD Falcon, USA) to obtain cell suspension. The cells were counted using a hemocytometer, and inoculated into 48-well culture plates at a density of 10^6^ cells/mL. Lymphocytes were cultured in RPMI-1640 medium containing 10% fetal calf serum (Hyclone) and 5 µg/mL concanavaline A (Sigma Aldrich) for 43 h, and then centrifuged (1000×*g*, 10 min). Supernatants were collected and preserved at – 80 °C until use. The levels of IL-4, IL-5, and IL-13 in the supernatants were measured using an ELISA (Boster, Wuhan, China), according to manufacturer’s instructions.

### Measurement of serum IgE level

Mice were sacrificed in a manner previously described [22]. Blood samples were collected and stored at room temperature for 1 h, and then centrifuged at 3000×*g* for 20 min. The supernatants were collected and stored at – 80 °C until use. IgE levels were measured by ELISA (Cusabio, Wuhan, China), according to manufacturer’s instructions.

### Analysis of bronchoalveolar lavage fluid (BALF)

BALF was collected and the total number of cells was counted using a hemocytometer. BALF was then centrifuged at 1000×*g* for 10 min. A cytospin sample was prepared and fixed with 4% paraformaldehyde for 30 min, and then the cells were stained with hematoxylin and eosin (H&E) (Solarbio, Beijing, China). Differential cell counts were observed under a light microscope. A total of 200 cells were counted (except for cell debris, cramps and abnormal looking cells), and the percentages of macrophages, lymphocytes, neutrophils, and eosinophils were determined.

### Histopathological examination of lung tissue

The left lungs were fixed in 4% formaldehyde, and then embedded in paraffin. Lung sections  (4 µm) were stained with H&E and periodic acid-Schiff (PAS) to identify airway inflammation and mucus production. Briefly, perivascular inflammation and peribronchial inflammation were used to quantify pulmonary inflammation. Airway inflammation was scored in a semi-quantitative manner as previously described [8]. Twenty sections of 10 mice from each group were examined, and at least 40 image fields at 200× magnification were viewed and scored. Each sample was assigned a random code so the examiner was not aware of the source of each section.

### Immunohistochemistry (IHC) evaluation of lung tissue

To examine lung tissue for RAGE, HDAC1, E-cadherin, and β-catenin, lung slices were deparaffinized and then submerged in citrate buffer (pH 6.0) at 95–100 ℃ for 15 min for antigen retrieval. The tissue slices were treated with H_2_O_2_ at room temperature for 15 min, and then incubated in recommended dilutions of primary antibodies at 4 ℃ overnight. Then the samples were incubated with secondary antibodies at room temperature for 20 min. The signals were displayed with DAB solution.

### Preparation of TDI-human serum albumin (TDI-HSA) conjugates

The preparation method of the TDI-HSA conjugate was described previously [23], which was a modification of the method described by Son [24]. Briefly, TDI was added to HSA in PBS with constant stirring. Then, the samples were centrifuged at 3000×*g* at room temperature for 20 min to remove unreacted TDI. The product was then dialyzed with PBS using cellulose membranes at 4 °C (Sigma Chemical Co., St. Louis, MO, USA) for 3 days.

### Culture, transfection, and treatment of cells

Human bronchial epithelial cell line 16HBE14o-(16HBE) cells (Shanghai Fuxiang Biological Technology Co., ATCC, Portland, OR, USA) were cultured in RPMI-1640 medium containing 10% fetal calf serum in an incubator with 5% CO_2_ at 37 °C. The cells passaged to five to eight generations were used for the following experiments. When 90% confluence was reached, the cells were passaged and inoculated to new culture plates, and various concentrations of TDI-HSA conjugate (0–100 µg/mL) were added to the culture medium and cells were cultured for different lengths of time. The TDI-HSA concentrations used were based on those used in prior study [25]. The lentiviral system used to knockdown RAGE was developed by Applied Biological Materials Inc (Nanjing, China). Lentiviral plasmids against RAGE proteins were constructed. These constructs and an empty vector were used to transfect 16HBE cells [10].

### Western blotting

In order to evaluate the expression of RAGE, HDAC1, AKT, p-AKT, E-cadherin, and β-catenin in vivo and in vitro, whole lung tissue and cell protein extracts were mixed with 5× SDS loading buffer. The samples were separated by 10% SDS–polyacrylamide gel electrophoresis and transferred to PVDF membranes (Millipore). Then the membranes were probed with anti-RAGE, anti-HDAC1, anti-AKT, anti-p-AKT, anti-E-cadherin, and anti-β-catenin antibodies using the recommended dilutions. The immunoreactive bands were imaged using an Odyssey® CLx Imager after incubation with an IRDye® 680WC-conjugated secondary antibody (LI-COR Biosciences). Odyssey software was used for data analysis, and Image J software was used for quantitative image analysis.

### Immunofluorescence microscopy

The localization of E-cadherin and β-catenin after stimulation with TDI-HSA was examined by immunofluorescence microscopy. 16HBE cells were cultured in RPMI-1640 containing 10% fetal calf serum on cell culture dishs (NEST, China), and were treated with 60 µg/mL TDI-HSA at 37 °C for 24 h with or without pretreatment of JNJ-26482585 (5 µM) or romidepsin (5 µM) for 2 h. Then, 16 HBE cells were fixed with 4% formaldehyde for 15 min at room temperature, treated with 0.5% Triton X-100 for 10 min, blocked with 5% BSA for 1 h, and then incubated with rabbit anti-E-cadherin and anti-β-catenin antibody at 4 °C overnight. The cells were incubated with Alexa Fluor 568-labeled donkey anti-rabbit IgG antibody for 1 h in the dark. The cells nuclei were stained with DAPI (Beyotime Biotechnology, China) for 10 min at room temperature. The distribution of E-cadherin and β-catenin was examined by laser-scanning confocal microscope (Zeiss, Germany).

### Statistical analysis

One-way analysis of variance (ANOVA) was used for comparison among groups, and Bonferroni post hoc test was used for multiple comparisons. Data were presented as mean ± standard deviation, and *P* < 0.05 was considered statistically significant. SPSS 20.0 software was used for statistical analysis.

## Results

### RAGE inhibitor reduced expression of HDAC1 and AKT phosphorylation, and ameliorated airway inflammation in TDI-induced asthmatic mice

**Fig. 1:**
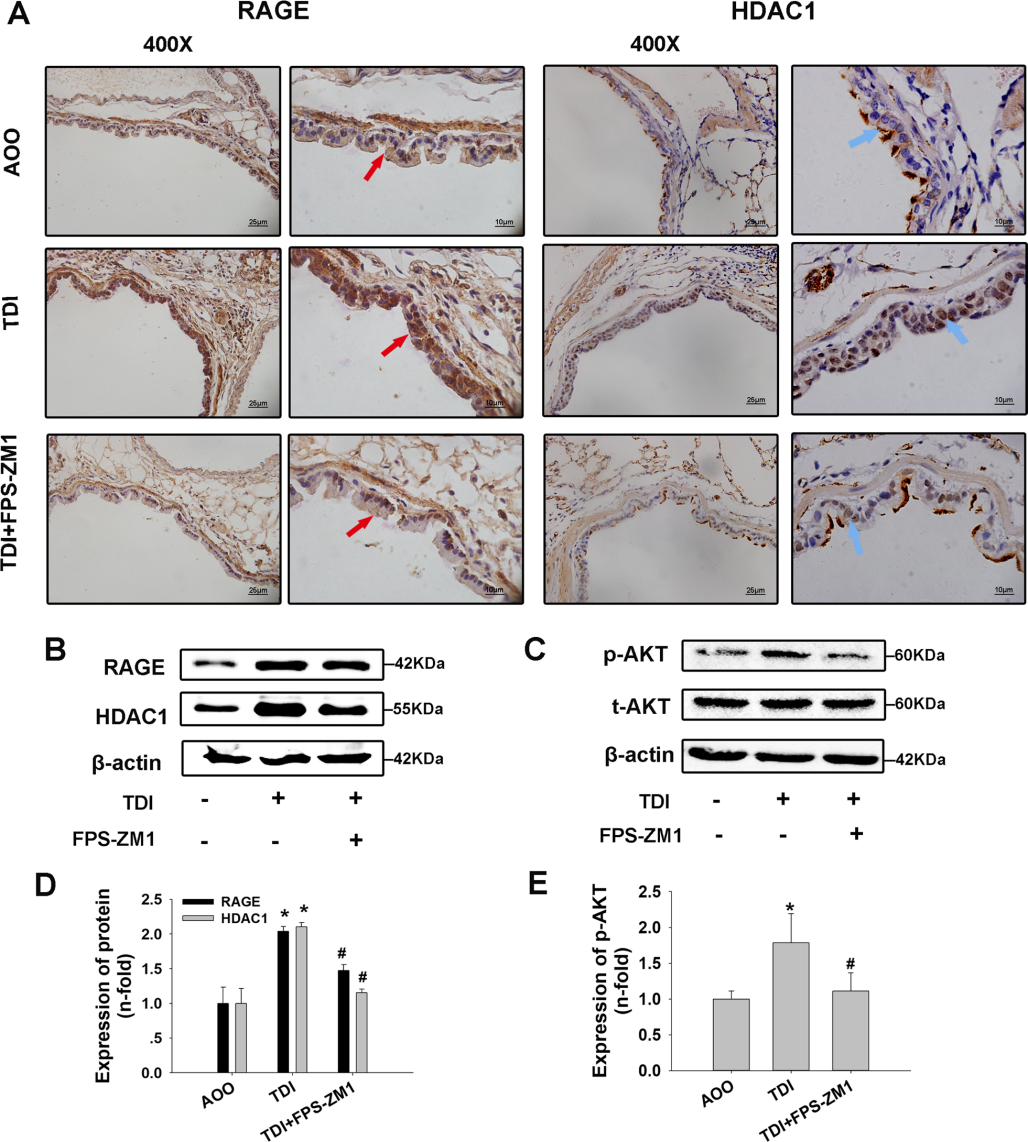
Effects of RAGE inhibitors on the expression of HDAC1 and p-AKT in TDI–induced asthmatic mice. **A** Immunohistochemistry analysis of the expression and distribution of RAGE and HDAC1 in airway epithelium. **B**, **C** Western blotting of RAGE, HDAC1, AKT and p-AKT expression in whole lung tissue. **D**, **E** Densitometric analysis of Western blots (n = 6). Red arrows indicate the expression of RAGE; Blue arrows indicate the expression of HDAC1. *P < 0.05 compared with AOO group; #*P* < 0.05 compared with TDI group

First, the expression of RAGE and HDAC1 was determined in each group. IHC staining showed increased expression of RAGE and HDAC1 in the TDI group. HDAC1 was mainly expressed in airway epithelium, with most of the immunostaining in the nucleus, while treatment with a RAGE inhibitor decreased HDAC1 expression (Fig. 1A). Western blotting showed the expression of RAGE and HDAC1 was upregulated in the TDI group, and significantly inhibited after blocking RAGE signaling with FPS-ZM1 (Fig. 1B, D). In addition, phosphorylation of AKT was upregulated after TDI exposure compared to AOO, indicating that the PI3K/AKT axis was activated. As expected, pretreatment with FPS-ZM1 attenuated these responses (Fig. 1C, E).

### Inhibition of HDAC reduced AHR and Th2 cytokines in TDI-induced asthmatic mice

**Fig. 2:**
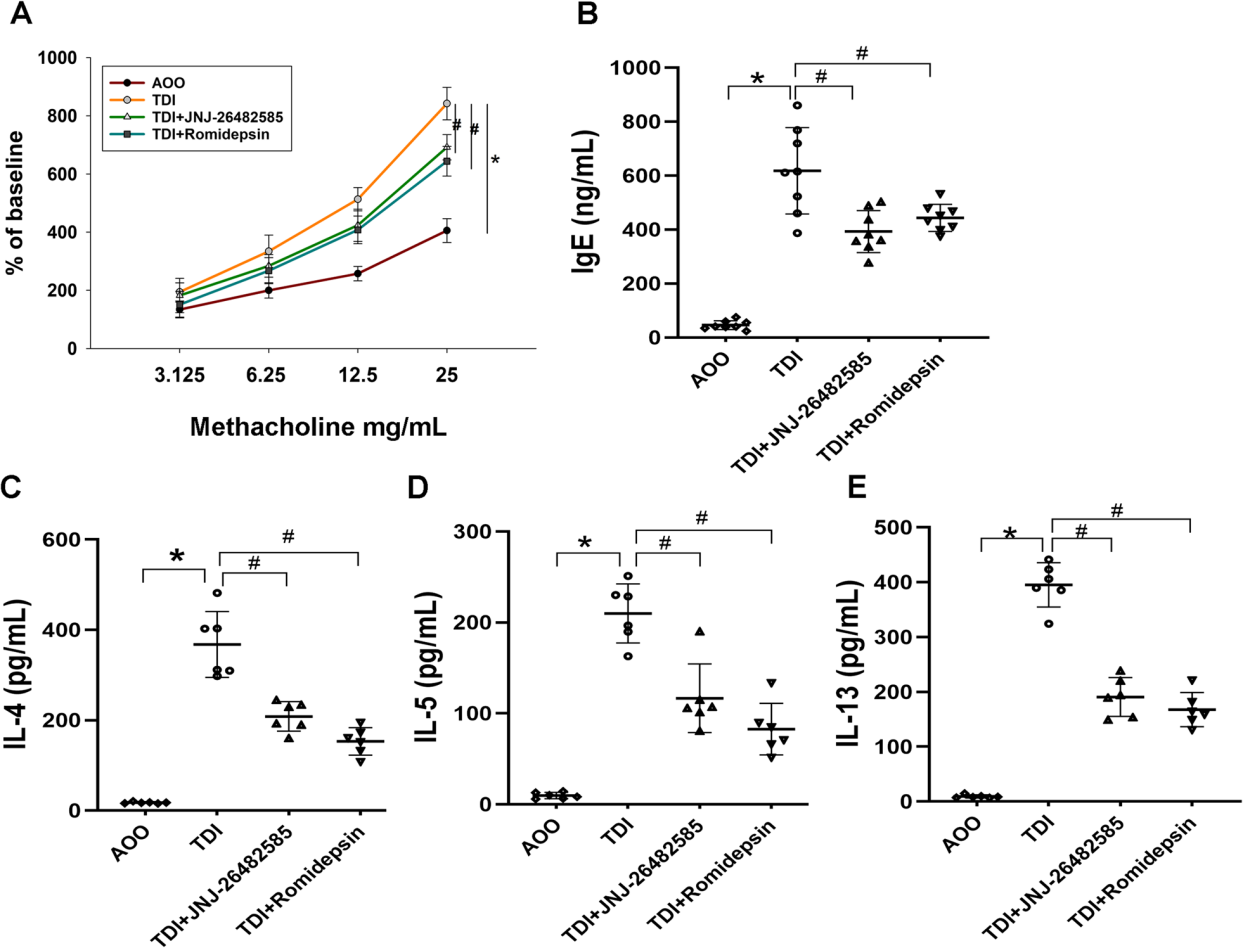
Effects of HDAC inhibitors on the asthmatic response in TDI-exposed mice. **A** Measurement of airway hyper-responsiveness by lung resistance (R_L_). Data were expressed as percentage of baseline value (n = 5). **B** Measurement of IgE in serum by ELISA. **C**–**E** Detection of IL-4, IL-5, and IL-13 in the supernatants of cultured lymphocytes by ELISA. *P < 0.05 compared with AOO group; #*P* < 0.05 compared with TDI group

To evaluate the effect of the HDAC pathway on TDI-induced asthma, AHR was measured 24 h after the last challenge. The results showed that AHR was significantly increased in TDI-induced mice challenged by methacholine compared with the AOO group (Fig. 2A). AHR was partially alleviated by intraperitoneal injection of an HDAC inhibitor, suggesting a prophylactic effect of HDAC inhibitors on TDI-induced AHR.

To evaluate the role of HDAC inhibitors in TDI-induced allergic airway inflammation, we examined the secretion of Th2 cytokines in the supernatants of cultured lymphocytes. The results indicated that JNJ-26482585 and romidepsin reduced levels of IL-4, IL-5 and IL-13 in the TDI group (Fig. 2C–E). Similarly, treatment with HDAC inhibitors markedly suppressed the increase in total serum IgE induced by TDI (Fig. 2B).

### HDAC inhibitors attenuated airway inflammation and goblet cell metaplasia in TDI-induced asthma

**Fig. 3:**
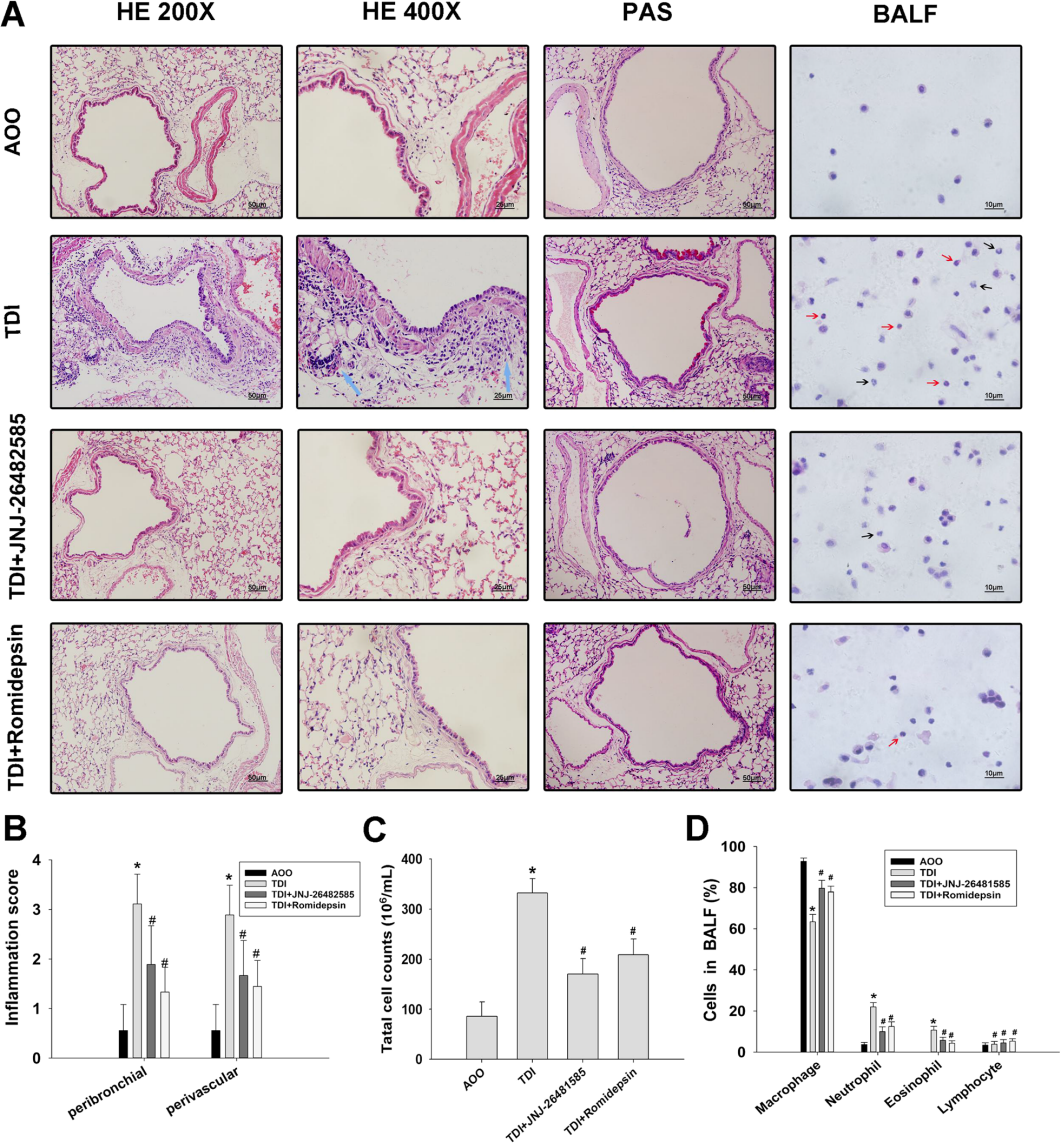
Effects of HDAC inhibitors on lung tissue of TDI-exposed asthmatic mice. **A** Representative lung tissue sections of each group stained with hematoxylin and eosin (H&E) at 200× and 400× magnification, and stained with PAS at 400× magnification. **B** Semi-quantitative analysis of airway inflammation. **C**, **D** Total inflammatory cell count and differential cell counts in the BALF of mice. A total of 200 cells stained with hematoxylin and eosin (H&E) in cytospun samples were counted to calculate the percentages of different inflammatory cells. n = 8–10. Blue arrows indicate inflammatory cells in the airways. Red arrows indicate infiltrating eosinophils; black arrows indicate infiltrating neutrophils. *P < 0.05 compared with the AOO group; #*P* < 0.05 compared with the TDI group

The total numbers and percentages of various inflammatory cells in BALF were determined. Consistent with total cell numbers (Fig. 3C), higher percentages of neutrophils and eosinophils were found in the TDI group, and these percentages were markedly decreased after administration of JNJ-26482585 and romidepsin (Fig. 3A, D). Examination of H&E stained tissues showed the typical pathological features of TDI-induced asthma; the bronchi were infiltrated by many inflammatory cells. Pretreatment of mice with JNJ-26482585 and romidepsin resulted in a significant reduction in perivascular and peribronchial inflammatory cell extravasation, and a decrease in the proliferation of airway epithelium (Fig. 3A, B). Examination of PAS-stained tissues showed goblet cell metaplasia in the TDI group, and pretreatment with JNJ-26482585 and romidepsin eliminated the development of metaplasia (Fig. 3A).

### HDAC inhibitors ameliorated redistribution of E-cadherin and β-catenin in TDI-induced asthma

**Fig. 4:**
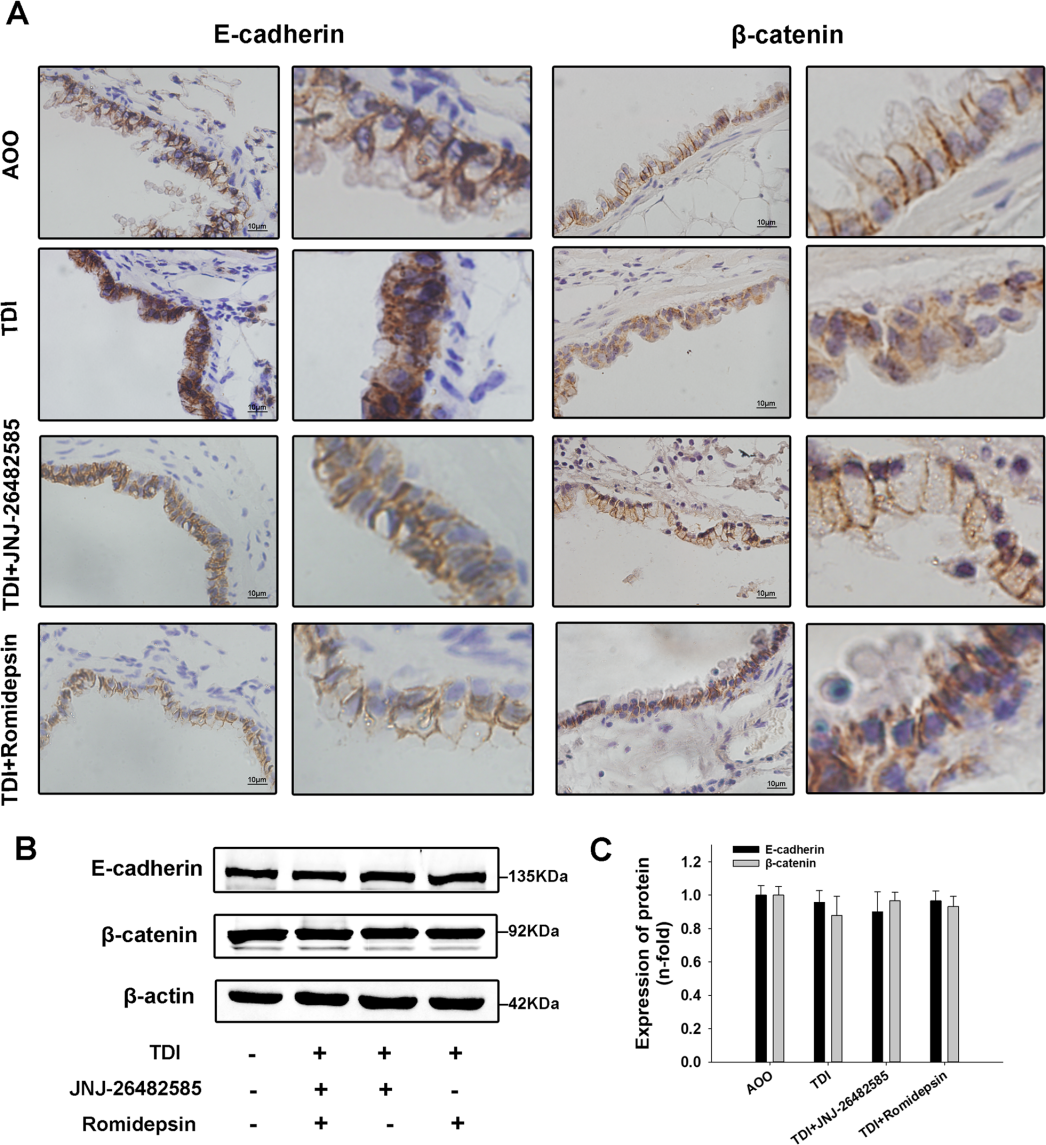
Effects of HDAC inhibitors on redistribution of E-cadherin and β-catenin in TDI-induced asthma. **A** Representative immunohistochemistry analysis of E-cadherin (400×) and β-catenin (400×), and amplified views of them in the lung. **B** Western blotting of E-cadherin and β-catenin expression in whole lung tissue. **C** Densitometric analysis of Western blots (n = 6)

The E-cadherin/β-catenin complex on the cell membrane is crucial in maintaining epithelial barrier integrity. Previous studies have shown that RAGE inhibition ameliorates the redistribution of the E-cadherin/β-catenin complex. In this study, we further detected the effect of HDAC inhibitors on the E-cadherin/β-catenin complex in TDI-induced asthma. Western blotting showed that HDAC inhibitors did not affect the expression of E-cadherin and β-catenin in TDI-induced asthma mice (Fig. 4B, C). Consistent with previous studies, E-cadherin and β-catenin were present in the lateral and apico-lateral borders of the airway epithelium in control mice, but were significantly reduced in the epithelial cell–cell contact areas and diffused in the cytoplasm and nucleus in the TDI-induced group. As expected, HDAC inhibitors ameliorated the redistribution of E-cadherin and β-catenin in TDI-induced asthmatic mice (Fig. 4A).

### RAGE blockade suppressed TDI-HSA-stimulated HDAC1 expression in 16HBE cells

**Fig. 5:**
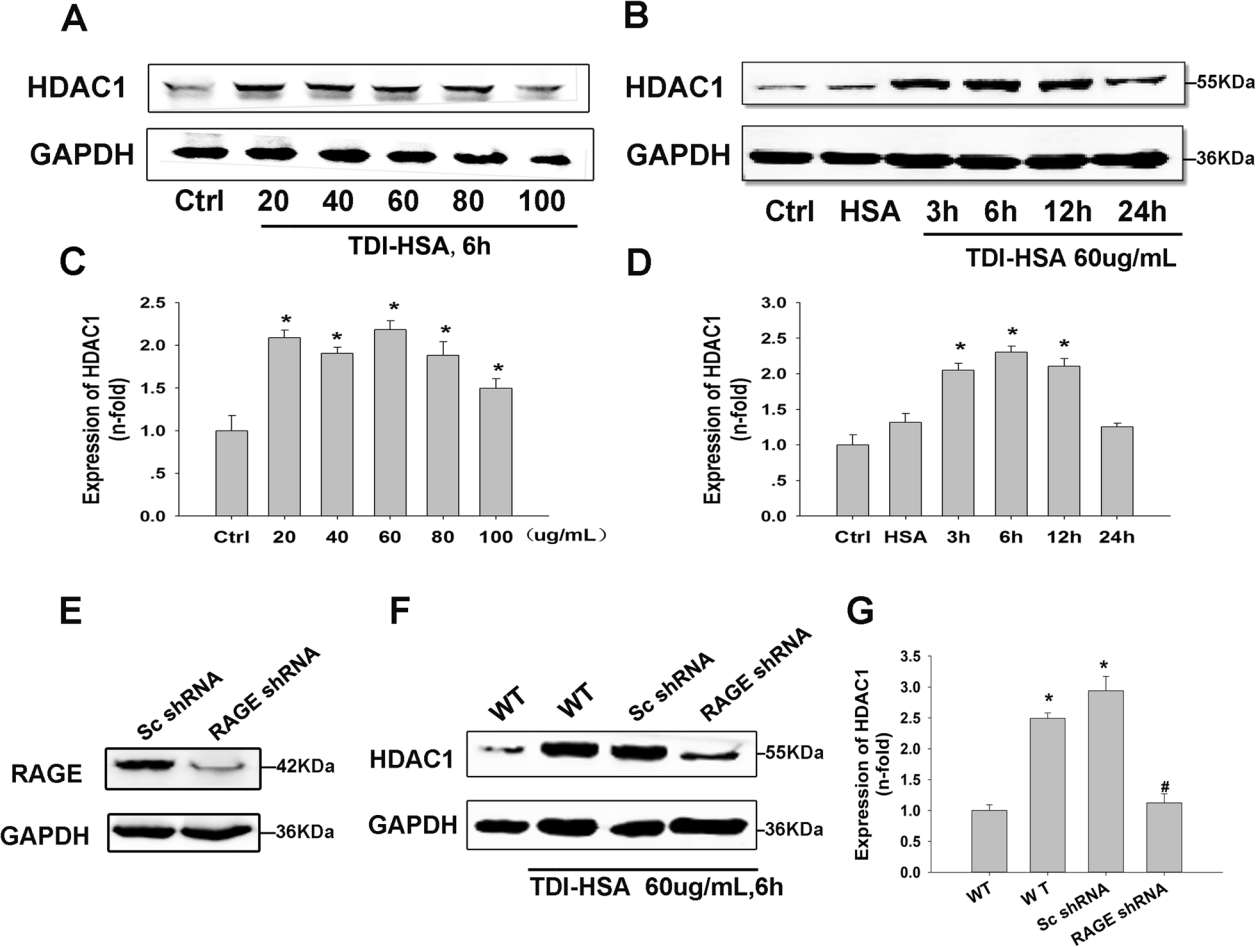
Effect of RAGE knockdown on the expression of HDAC1 stimulated by TDI-HSA in 16HBE cells. **A** 16HBE cells were stimulated with various concentrations of TDI-HSA (0, 20, 40, 80, 100 µg/mL) for 6 h, and the expression of HDAC1 was detected. **B** 16HBE cells were stimulated with 60 µg/mL TDI-HSA for indicated times, and the expression of HDAC1 was determined. **E** Verification of RAGE knockdown in 16HBE cell via Western blotting. **F** Effect of TDI-HSA stimulation (60 µg/mL) for 6 h on the expression of HDAC1 in 16HBE cells. **C**, **D**, **G** Desitometric analysis of Western blots (n = 3). *P < 0.05 versus control. #*P* < 0.05 compared with the Sc shRNA group

Previous studies have confirmed that RAGE expression was markedly increased in 16HBE cells after TDI-HSA stimulation [10], so we continued using 16HBE in the present study. The expression of HDAC1 in 16HBE cells was examined after stimulation with TDI-HSA. Western blotting revealed HDAC1 expression was markedly increased after TDI-HSA stimulation (Fig. 5A), and this effect peaked between 3 and 12 h after TDI-HSA stimulation (Fig. 5B). In order to identify whether RAGE signaling affects the upregulation of HDAC1 stimulated by TDI-HSA in vitro, 16HBE cells were transfected with lentiviral-expressed RAGE shRNA and then selected using a proper concentration of puromycin (Fig. 5E). 16HBE cells with RAGE-shRNA were stimulated with 60 μg/mL TDI-HSA for 6 h. and silencing RAGE markedly inhibited the increased expression of HDAC1. These findings suggest that RAGE may be involved in regulating HDAC1 in 16HBE cells (Fig. 5F, G).

### Inhibition of the PI3K/AKT axis suppressed the increased expression of HDAC1 induced by TDI-HSA in 16HBE cells

**Fig. 6:**
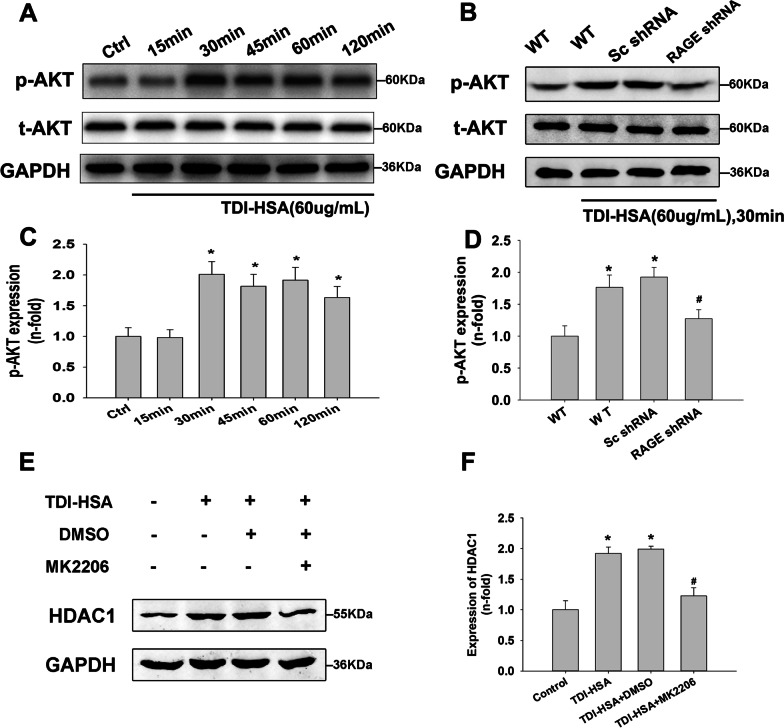
Effects of AKT pathway intervention on HDAC1 expression induced by TDI-HSA. **A** Western blotting revealed that the phosphorylation of AKT was increased by TDI-HSA stimulation (60 µg/mL). **B** Western blotting showed that RAGE knockdown decreased the expression of p-AKT induced by TDI-HSA in 16HBE cells. **E** Western blotting revealed that AKT inhibition decreased TDI-HSA induced expression of HDAC1 in 16HBE cells. **C**, **D**, **F** Desitometric analysis of Western lots (n = 3). *P < 0.05 versus control. #*P* < 0.05 compared with the Sc shRNA group (**D**) and TDI-HSA + DMSO group (**F**)

We detected the relevant signaling pathways to determine the mechanism by which RAGE mediates the TDI-induced upregulation of HDAC1, Consistent with in vivo experiments, PI3K/AKT signaling was activated in 16HBE cells in response to TDI-HSA stimulation (Fig. 6A). Western blotting showed that AKT phosphorylation was upregulated, and this upregulation was inhibited by RAGE silencing (Fig. 6B). To further elucidate the involvement of the PI3K/AKT pathway in regulating HDAC1, 16HBE cells were treated with an AKT inhibitor (MK2206, 1 µM) prior to TDI-HSA stimulation. Pretreatment with MK2206 inhibited the TDI-induced upregulation of HDAC1 (Fig. 6C). These results suggest that RAGE may regulate HDAC1 expression through the PI3K/AKT pathway.

### HDAC inhibition ameliorated TDI-HSA-induced redistribution of E-cadherin and β-catenin in 16HBE cells

**Fig. 7:**
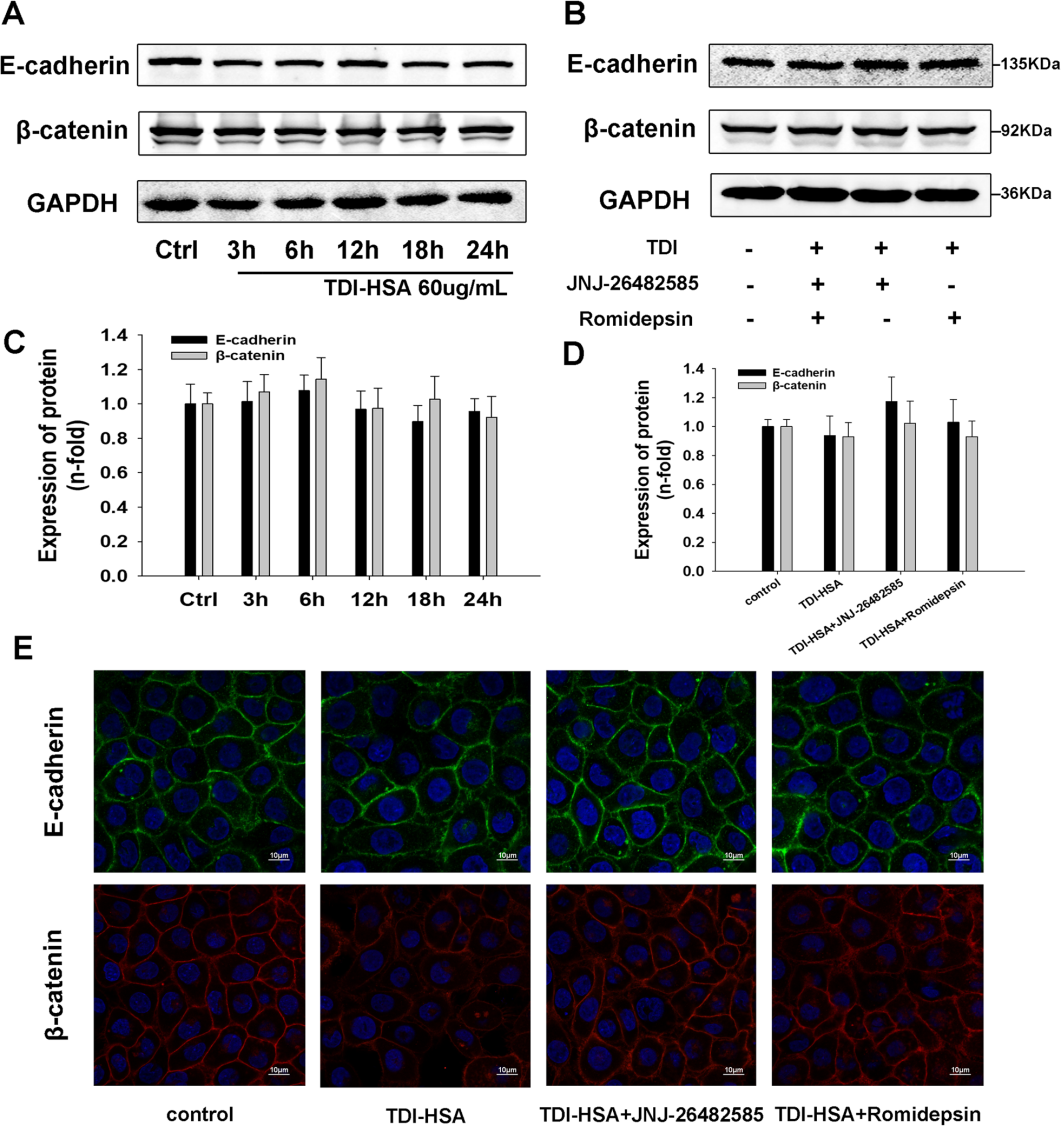
Effects of HDAC inhibitors on TDI-HSA-induced redistribution of E-cadherin and β-catenin in 16HBE cells. **A** 16HBE cells were stimulated with 60 µg/mL TDI-HSA for indicated times to examine the expression of E-cadherin and β-catenin. **B** 16HBE cells were stimulated with TDI-HSA (60 µg/mL) for 24 h with or without pretreatment with JNJ-26482585 (5 µM) and romidepsin (5 µM) for 2 h. Western blotting revealed that HDAC inhibitors did not affect the expression of E-cadherin or β-catenin in 16HBE cells. **C**, **D** Densitometric analysis of Western blots (n = 3). **E** Immunofluorescence staining was used to visualize the localization of E-cadherin and β-catenin

The expression of E-cadherin and β-catenin in 16HBE cells was examined after stimulation with 60 µg/mL TDI-HSA for various times. Western blotting showed that E-cadherin and β-catenin expression in 16HBE cells were unaffected by TDI-HSA treatment (Fig. 7A, B). To identify the effect of HDAC inhibitors on the redistribution of E-cadherin and β-catenin, cells were pretreated with JNJ-26482585 (5 µM) and romidepsin (5 µM) for 2 h, and then stimulated by TDI-HSA (60 µg/mL) for 24 h. Western blotting showed that HDAC inhibitors did not affect the expression of E-cadherin and β-catenin in 16HBE cells. The immunofluorescence results showed that TDI-HSA promoted the delocalization of E-cadherin and β-catenin in 16HBE cells, At the same time, HDAC inhibition ameliorated the redistribution of E-cadherin and β-catenin in the TDI-HSA group, indicating that HDAC may play a vital role in TDI-HSA induced airway epithelial barrier disruption.

## Discussion

In this study, we found for the first time that RAGE is a potential positive regulator of HDAC1 in a TDI-induced asthma model, and its role may be related to the PI3K/AKT signaling pathway. We also demonstrated that inhibition of HDAC prevented TDI-induced airway inflammation prophylactically.

RAGE has been shown to play a vital role in asthma using several types of asthma models. Recently, Perkins et al. reported that RAGE is a critical component of type 2 cytokine signaling, which is a driving force in the pathogenesis of type 2 asthma [26]. Consistent with a previous study, the TDI-induced mice in this study exhibited characteristic features of asthma, including AHR, Th2 responses and airway inflammation, which were attenuated by treatment with FPS-ZM1 ^8,9^. However, the exact mechanism by which RAGE mediates TDI-induced airway inflammation has yet to determined.

Subtype HDAC1 has been shown to play a vital role in asthma. Wawrzyniak et al. observed that HDAC1 expression was significantly increased in human bronchial epithelial cells (HBECs) of asthmatic patients [16]. Similarly, another study reported that the single nucleotide polymorphism (SNP) of HDAC1 (rs1741981) was significantly associated with asthma severity and response to corticosteroids [15]. HDAC1 activity in lung tissue of OVA-induced asthmatic mice was greater than that in the lung tissue of control mice [27]. A previous study showed that administration of a RAGE agonist, such as vascular endothelial growth factor (VEGF) or glycated-albumin, increased HDAC activity in the retinal pigment epithelium [21]. Sundar et al. reported that electronic cigarettes caused a decrease in HDAC2 in gingival epithelial cells via a RAGE-dependent pathway [28]. In this study, we found that expression of HDAC1 was increased in TDI-induced asthmatic mice, and the increase was attenuated by inhibiting RAGE signaling with FPS-ZM1, indicating that RAGE may regulate airway inflammation via HDAC1.

Consistent with previous studies, we found that p-AKT expression were significantly upregulated in whole lung tissue and airway epithelium in the TDI group compared to the control group [29, 30], and this upregulation was blocked when mice were pretreated with FPS-ZM1. Woo SR et al. reported that NANOG, a transcription factor for tumor cells, promotes HDAC1 protein stabilization through the AKT signaling pathway [31]. Choi Y S et al. found that PI3K and PDK1 pathways can affect NF-kB function by regulating HDAC1 [32]. In this study, we found that pretreatment with the AKT inhibitor MK2006 suppressed TDI-induced upregulation of HDAC1 in 16HBE cells. Collectively, these results indicate that RAGE may regulate HDAC1 expression in TDI-induced asthma via the PI3K/AKT pathway. Other studies using experimental models have also indicated that the PI3K/AKT signaling pathway is involved in the regulation of other HDAC subtypes [33, 34].

To further examine the relationship between HDAC1 and asthma, TDI-induced asthmatic mice were treated with JNJ-26482585 and romidepsin, relatively specific inhibitors of HDAC1. Treatment with HDAC inhibitors alleviated the increase in AHR, as measured by non-invasive lung function in this study. It would be more persuasive if invasive lung function was used. In addition, inhibitors of HDACs attenuated the airway inflammatory response, and the increase in Th2 cytokines. Ren et al. reported that HDAC inhibition suppressed airway remodeling, AHR, and airway inflammation in an OVA-exposed asthma model [17]. Another study demonstrated that treatment with HDAC inhibitors suppressed airway inflammation in a murine model of chronic allergic airway disease [35]. The current findings indicate that blocking HDAC activity may be a novel therapeutic intervention for asthmatic patients. However, it is important to develop more specific inhibitors of HDACs.

The respiratory epithelium provides a physical and immunological barrier that protects the host from the potential hazards of inhaled environmental factors [36]. There is growing evidence that an abnormal airway epithelial barrier plays a vital role in sensitization to allergenss associated with the onset of asthma [36, 37]. Disruption of epithelial barrier and loss of the junctional proteins E-cadherin and β-catenin in the cytomembrane were observed in TDI-induced asthmatic mice, a finding consistent with previous studies [8]. As expected, HDAC inhibitors ameliorated the redistribution of E-cadherin and β-catenin in TDI-induced asthmatic mice. Other studies have demonstrated that blocking HDAC activity may be a novel target for improving epithelial barrier function in asthma and allergic rhinitis [16, 38]. Besides, HDAC3 inhibitors regulate the expression of pro-inflammatory genes by selectively regulating the acetylation status of the transcription factor NF-κB in macrophages [39]. Colley T et al. found that SIRT 1 is involved in the acetylation of the transcription factor GATA 3 in Th2 cells, thereby regulating the expression of Th2-related cytokines [40]. Another study demonstrated that HDAC inhibitors promote Th17 differentiation by increasing the expression of the regulatory transcription factor RORγT [41]. Further studies are needed to explore whether HDAC inhibitors affect other immune cells in the TDI asthma model.

In conclusion, our results suggest that RAGE modulates HDAC1 expression via the PI3K/AKT pathway, and that inhibition of HDAC prevents TDI-induced airway inflammation. These results help understand how RAGE mediates airway inflammation, and may provide insights for novel asthma treatments.

## Supplementary Information

**Additional file 1: Fig S1**. Original results of western blot assays in the laboratory

## Data Availability

The data set during and/or analyzed during the current study available from the corresponding author on reasonable request.

## Abbreviations

TDI: Toluene diisocyanate
RAGE: Receptor for advanced glycation end products
HDAC: Histone deacetylase
16HBE: Human bronchial epithelial cell line 16HBE14o-
AHR: Airway hyperresposiveness
R_L_: Lung resistance
BALF: Bronchoalveolar lavage fluids
DAPI: 4′,6-Diamidino-2-phenylindole dihydrochloride
One-way ANOVA: One-way analysis of variance
