# Supplementary material for: RAGE mediates airway inflammation via the HDAC1 pathway in a toluene diisocyanate-induced murine asthma model
Source: BMC Pulm Med. 2022 Feb 11;22:61. doi: 10.1186/s12890-022-01832-3 (PMC8832863; doi:10.1186/s12890-022-01832-3)
Supplement: Supplementary file 1 — Additional file 1: Fig S1. Original results of western blot assays in the laboratory [file 12890_2022_1832_MOESM1_ESM.pdf]

Fig S1. Original results of western blot assays in the laboratory.

Fig IB

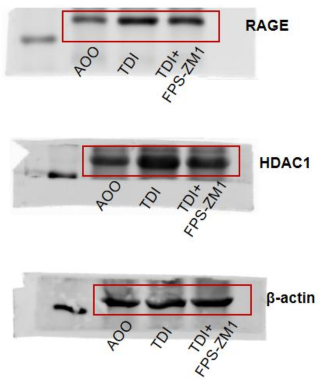

Fig IC

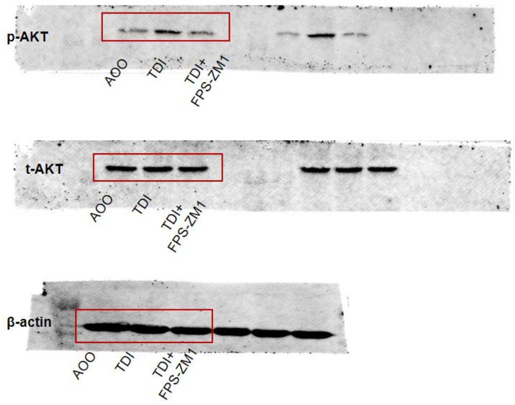

Fig 4B

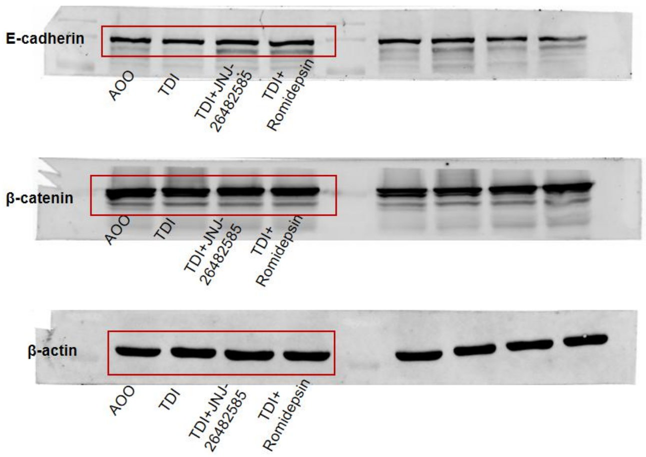

Fig 5A

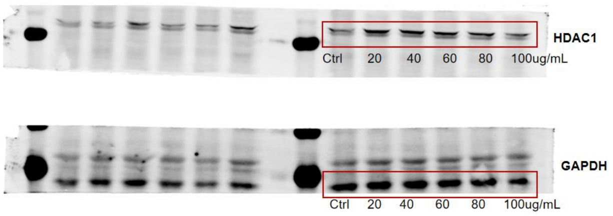

Fig 5B

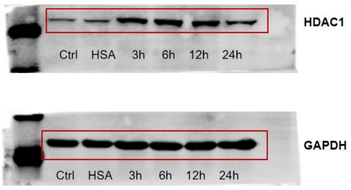

Fig 5E

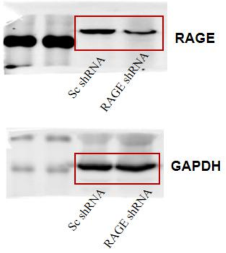

Fig 5F

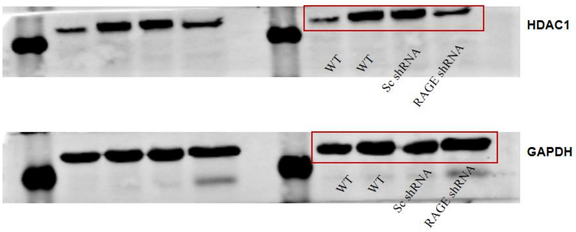

Fig 6A

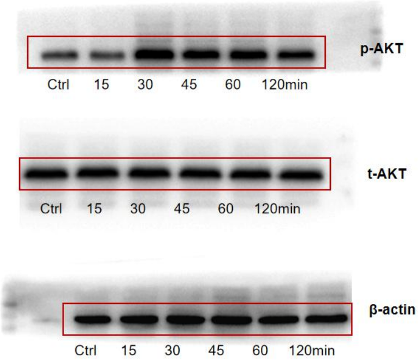

Fig 6B

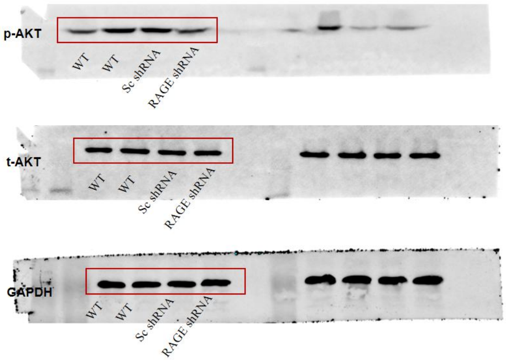

Fig 6E

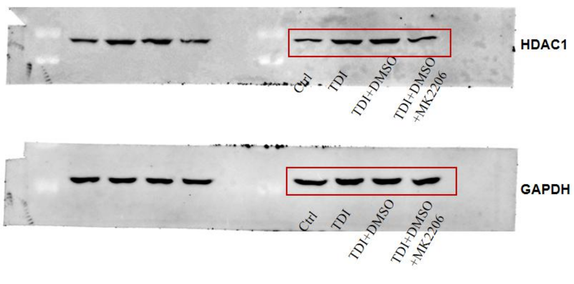

Fig 7A

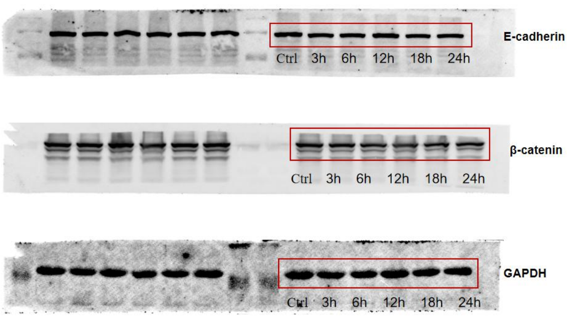

E-cadherin
Ctrl 3h 6h 12h 18h 24h
β-catenin
Ctrl 3h 6h 12h 18h 24h
GAPDH
Ctrl 3h 6h 12h 18h 24h

Fig7B

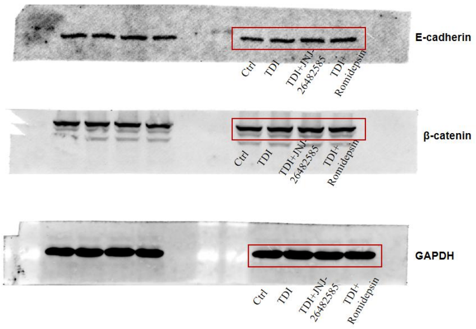

E-cadherin
Ctrl
TDI
TDI+JNJ-26482585
TDI+ Romidepsin
β-catenin
Ctrl
TDI
TDI+JNJ-26482585
TDI+ Romidepsin
GAPDH
Ctrl
TDI
TDI+JNJ-26482585
TDI+ Romidepsin
